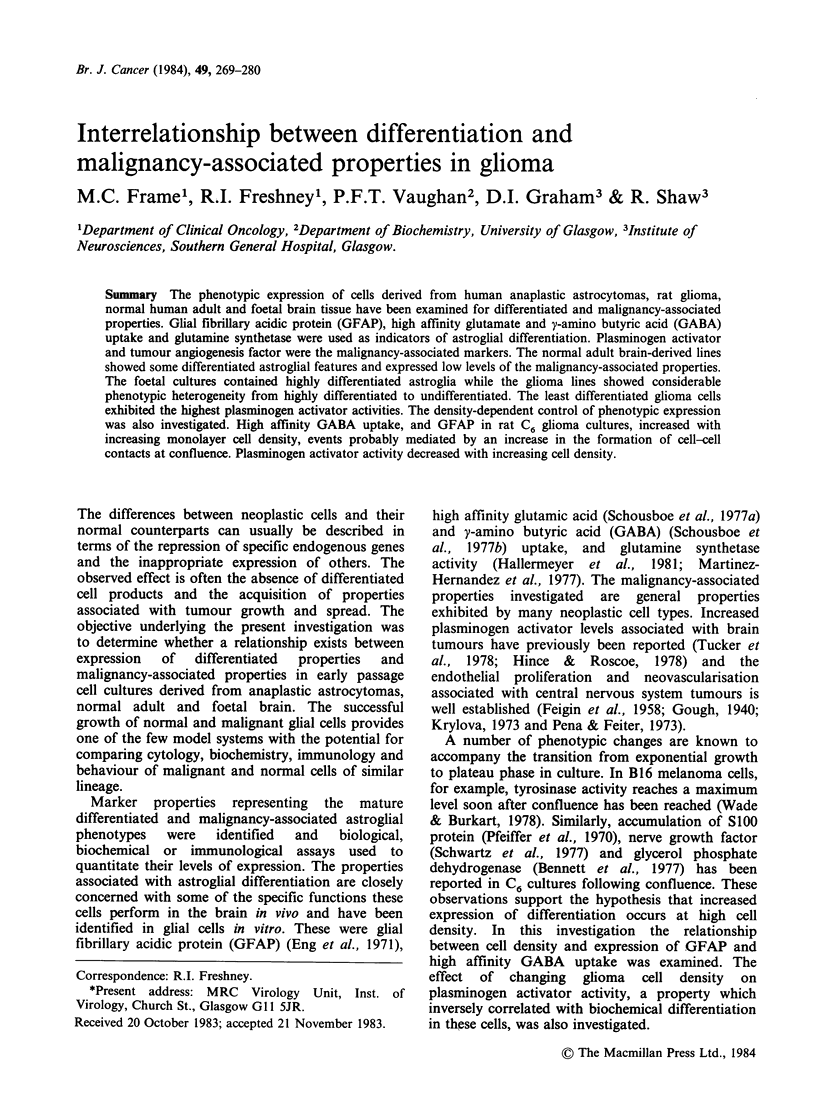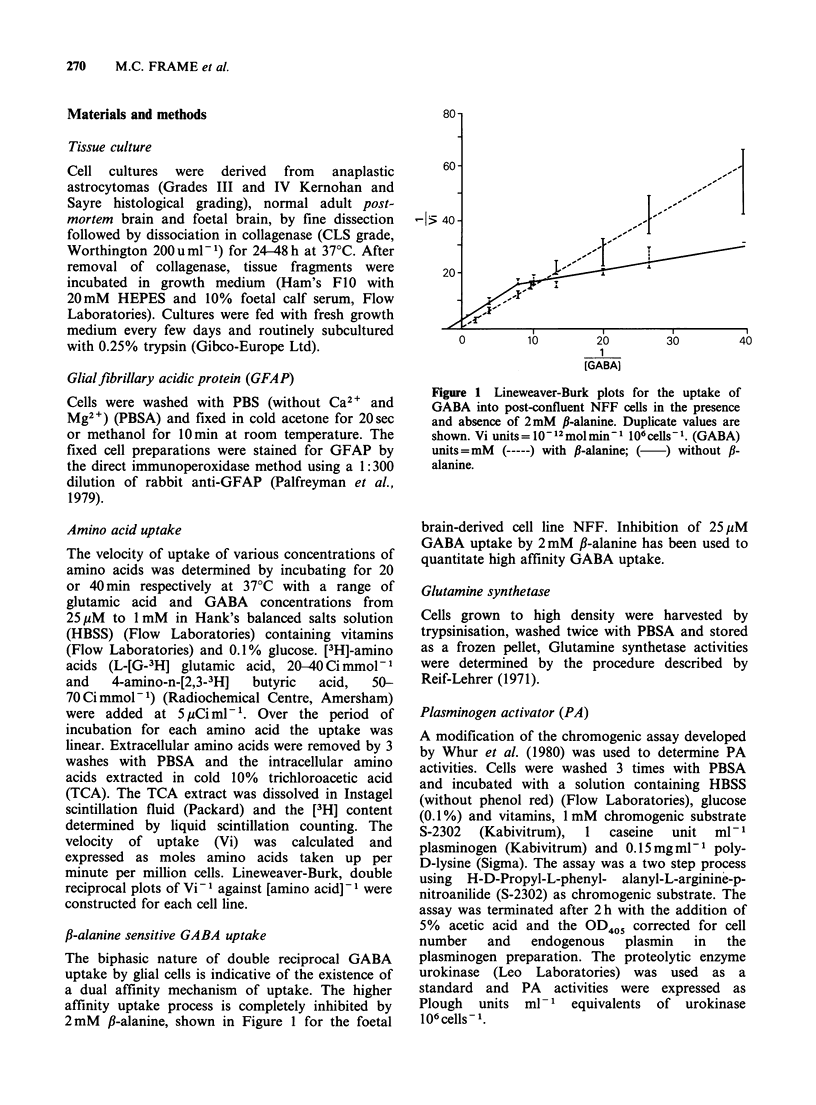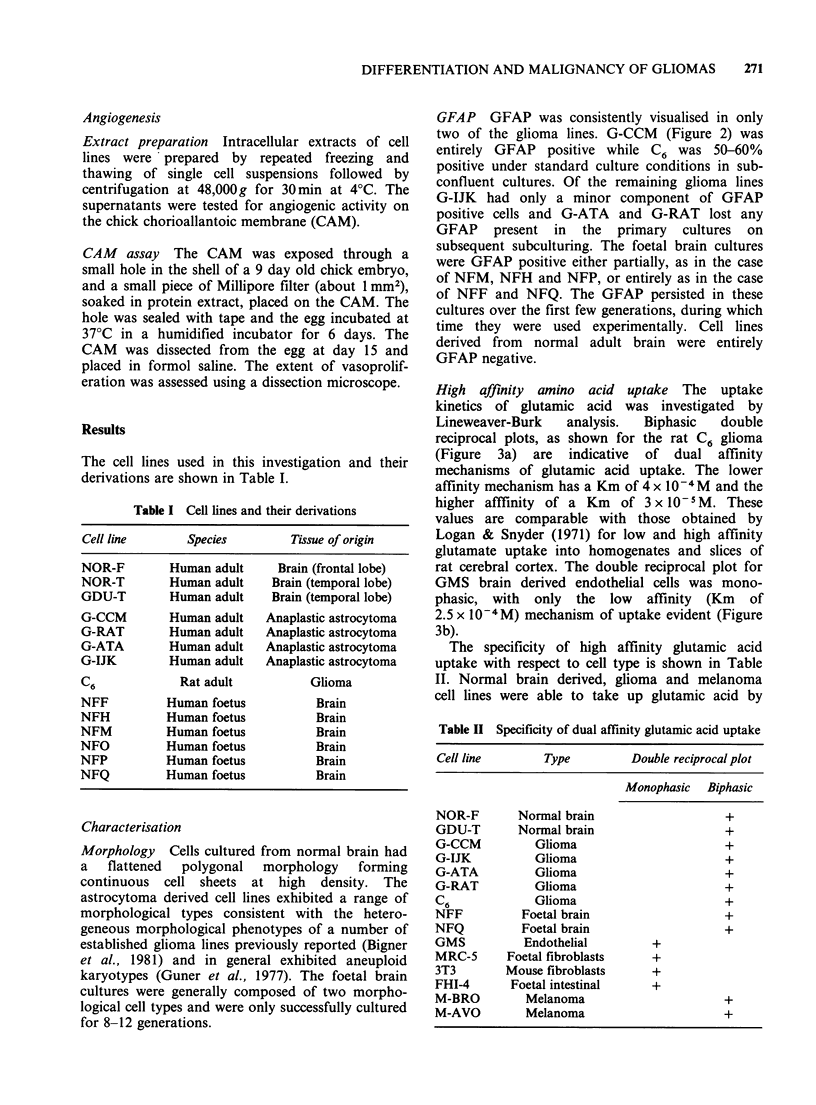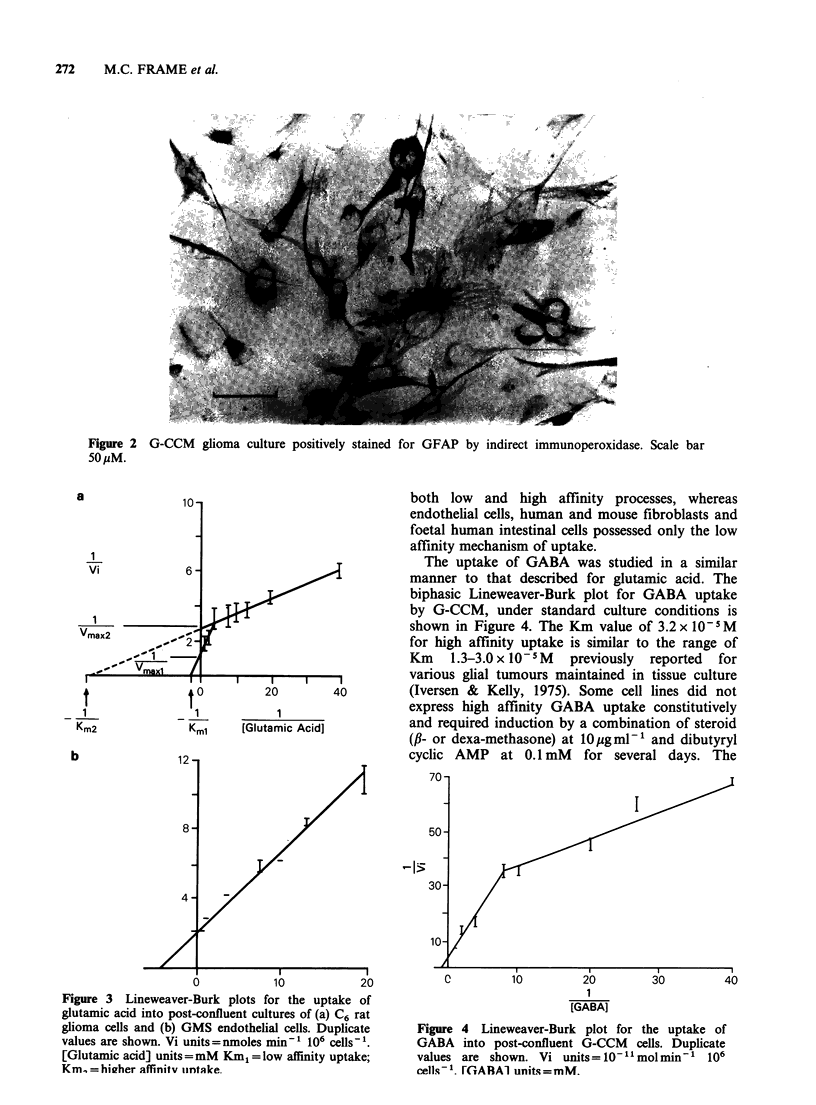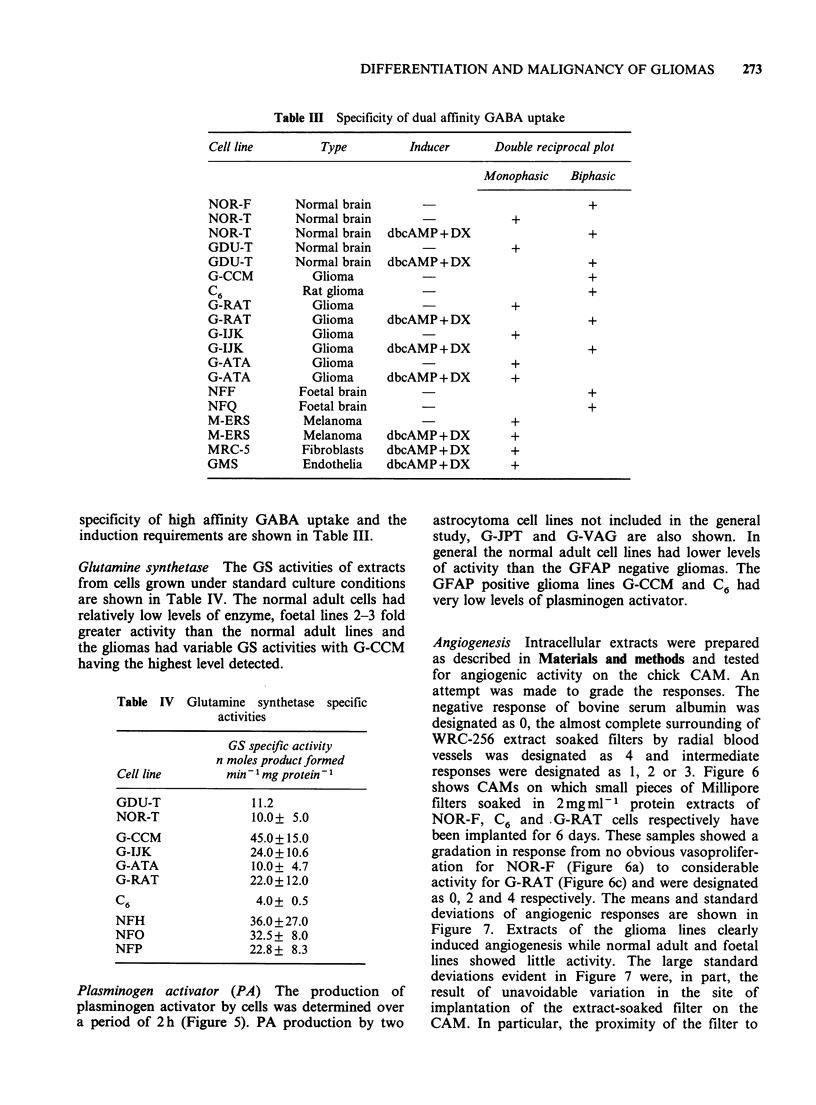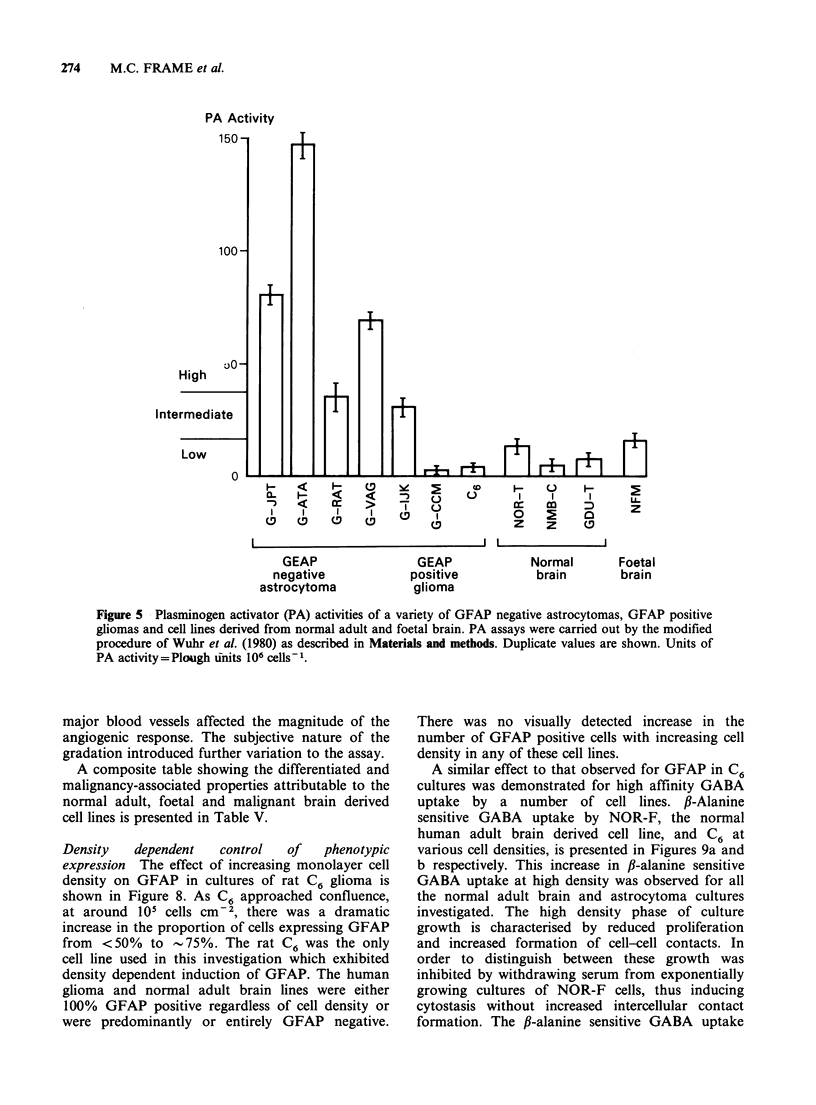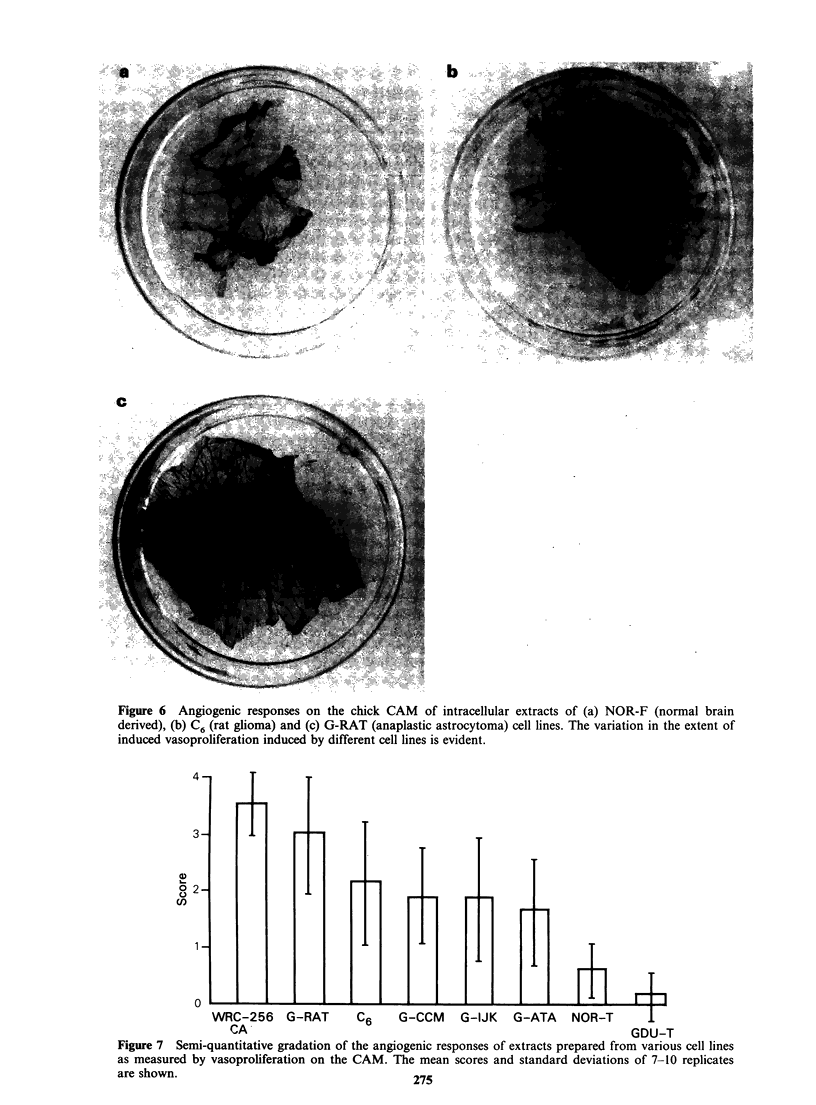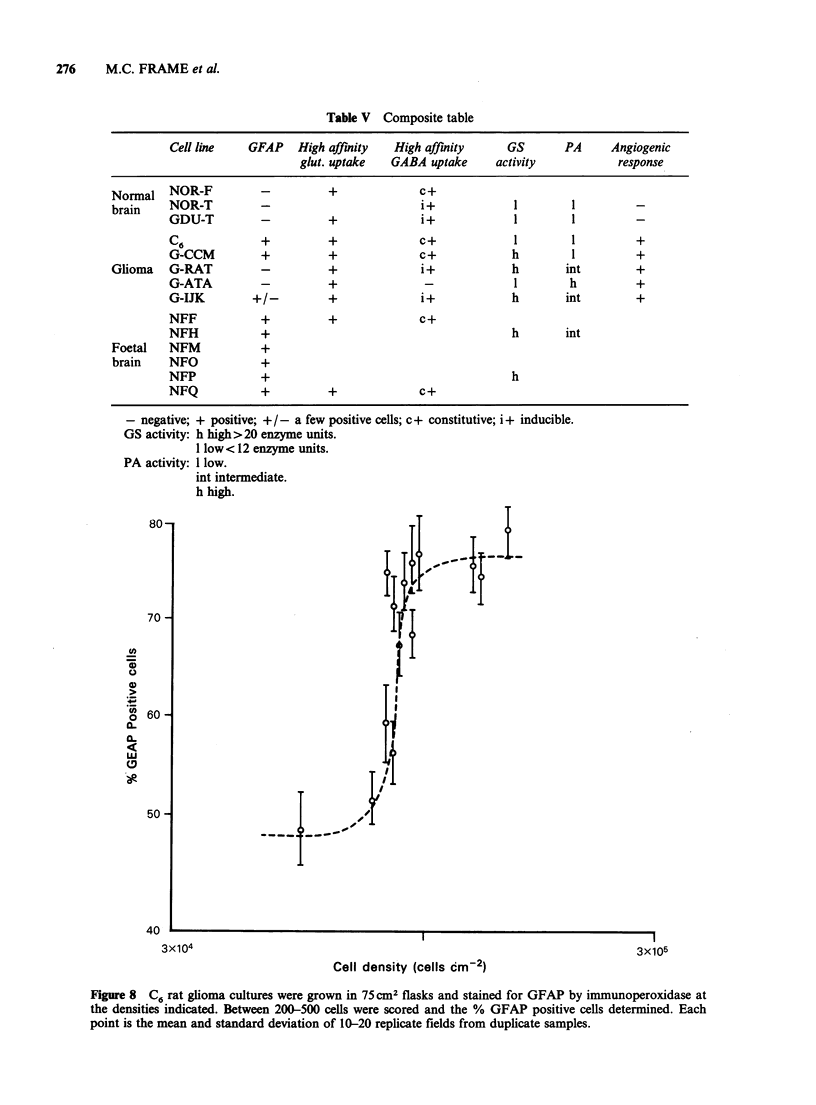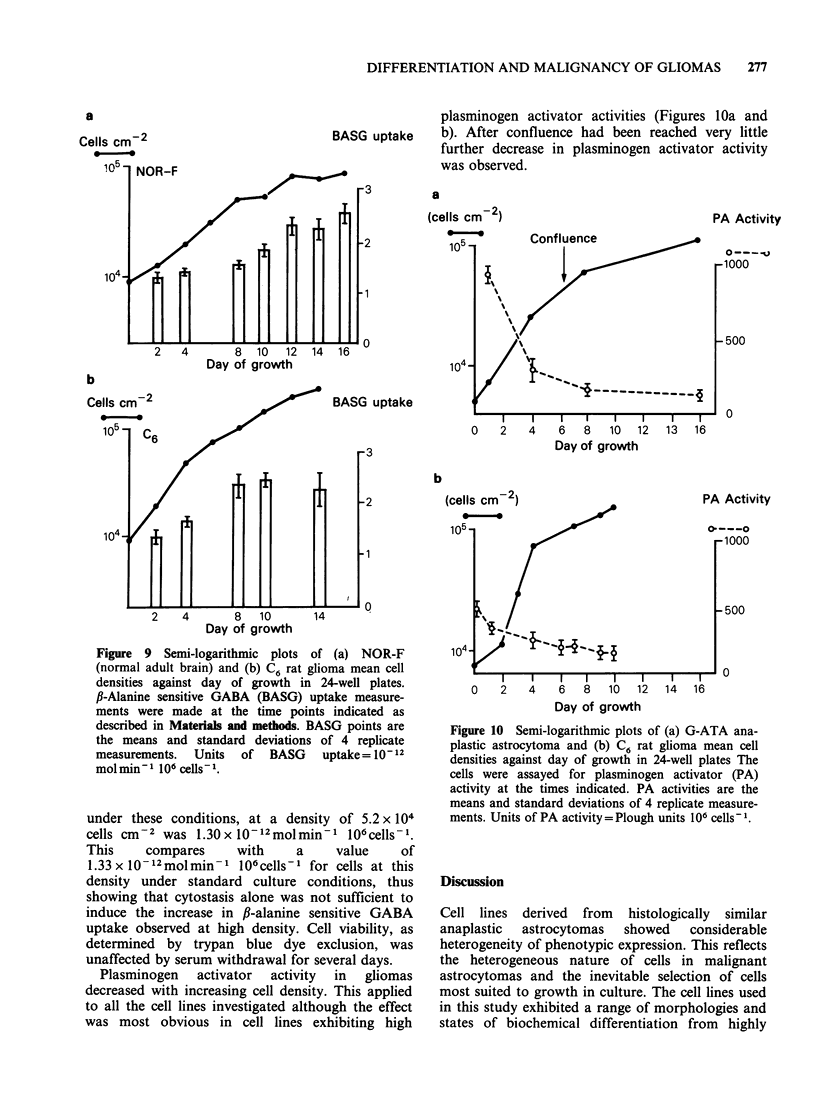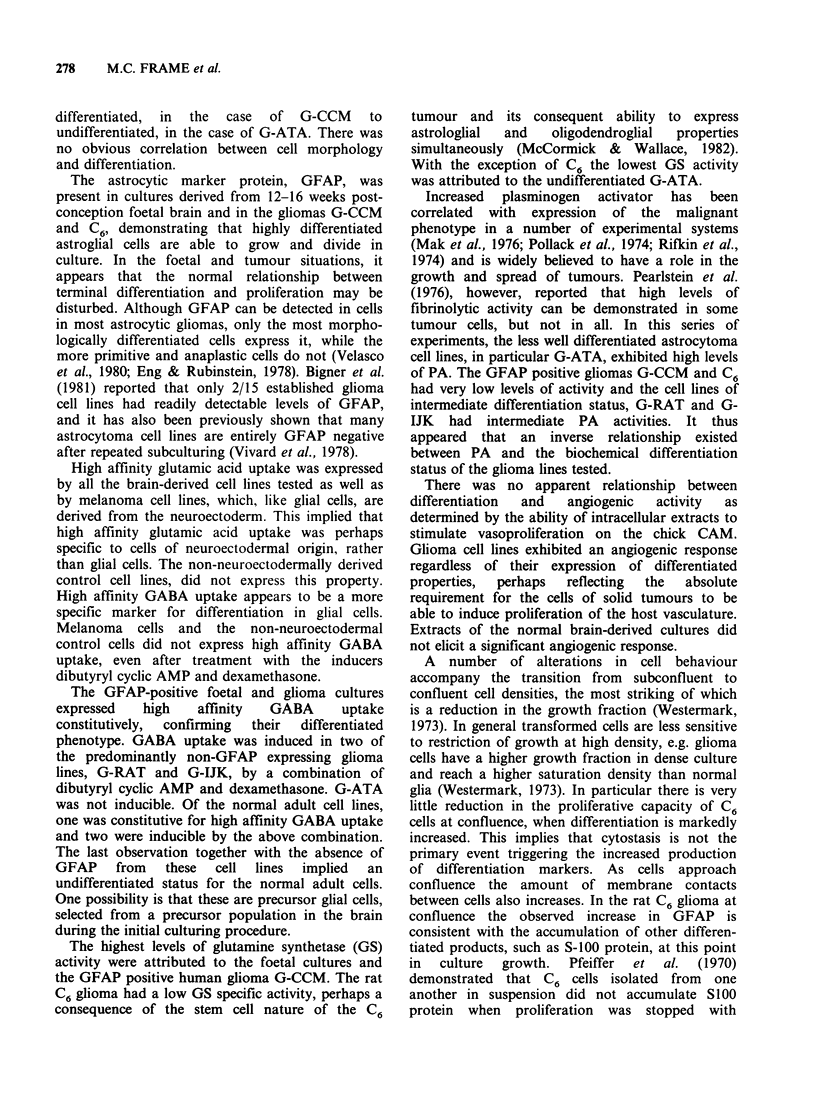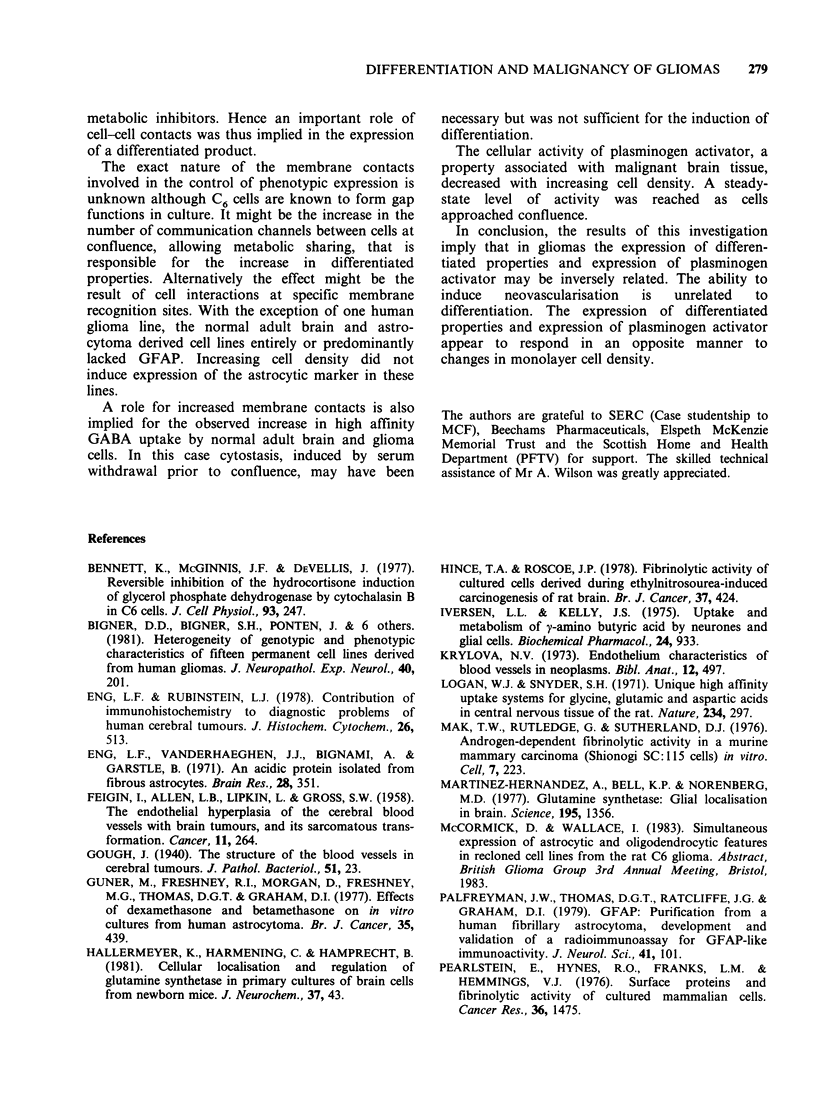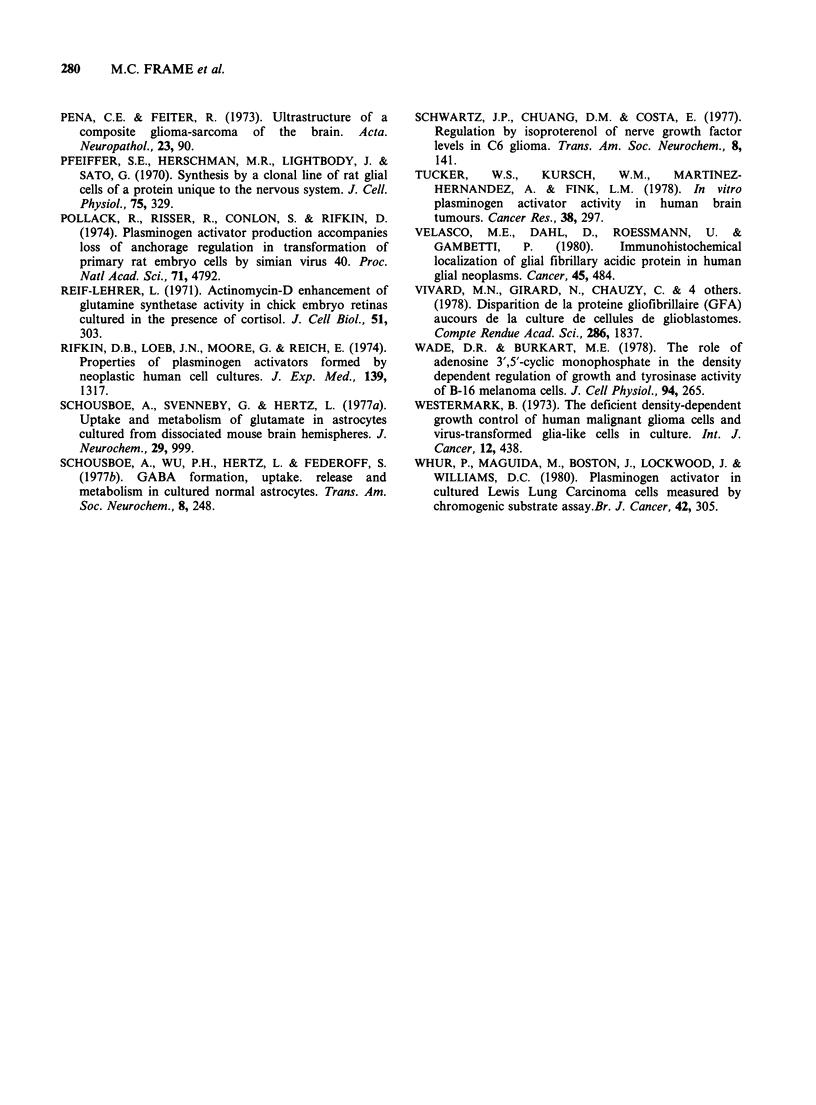# Interrelationship between differentiation and malignancy-associated properties in glioma.

**DOI:** 10.1038/bjc.1984.44

**Published:** 1984-03

**Authors:** M. C. Frame, R. I. Freshney, P. F. Vaughan, D. I. Graham, R. Shaw

## Abstract

**Images:**


Br. J. Cancer (1984), 49, 269–280

### Interrelationship between differentiation and malignancy-associated properties in glioma

M.C. Frame[1], R.I. Freshney[1], P.F.T. Vaughan[2], D.I. Graham[3] & R. Shaw[3]

[1]*Department of Clinical Oncology,* [2]*Department of Biochemistry, University of Glasgow,* [3]*Institute of Neurosciences, Southern General Hospital, Glasgow.*

**Summary** The phenotypic expression of cells derived from human anaplastic astrocytomas, rat glioma, normal human adult and foetal brain tissue have been examined for differentiated and malignancy-associated properties. Glial fibrillary acidic protein (GFAP), high affinity glutamate and γ-amino butyric acid (GABA) uptake and glutamine synthetase were used as indicators of astroglial differentiation. Plasminogen activator and tumour angiogenesis factor were the malignancy-associated markers. The normal adult brain-derived lines showed some differentiated astroglial features and expressed low levels of the malignancy-associated properties. The foetal cultures contained highly differentiated astroglia while the glioma lines showed considerable phenotypic heterogeneity from highly differentiated to undifferentiated. The least differentiated glioma cells exhibited the highest plasminogen activator activities. The density-dependent control of phenotypic expression was also investigated. High affinity GABA uptake, and GFAP in rat $C_6$ glioma cultures, increased with increasing monolayer cell density, events probably mediated by an increase in the formation of cell–cell contacts at confluence. Plasminogen activator activity decreased with increasing cell density.

The differences between neoplastic cells and their normal counterparts can usually be described in terms of the repression of specific endogenous genes and the inappropriate expression of others. The observed effect is often the absence of differentiated cell products and the acquisition of properties associated with tumour growth and spread. The objective underlying the present investigation was to determine whether a relationship exists between expression of differentiated properties and malignancy-associated properties in early passage cell cultures derived from anaplastic astrocytomas, normal adult and foetal brain. The successful growth of normal and malignant glial cells provides one of the few model systems with the potential for comparing cytology, biochemistry, immunology and behaviour of malignant and normal cells of similar lineage.

Marker properties representing the mature differentiated and malignancy-associated astroglial phenotypes were identified and biological, biochemical or immunological assays used to quantitate their levels of expression. The properties associated with astroglial differentiation are closely concerned with some of the specific functions these cells perform in the brain *in vivo* and have been identified in glial cells *in vitro*. These were glial fibrillary acidic protein (GFAP) (Eng *et al.*, 1971), high affinity glutamic acid (Schousboe *et al.*, 1977*a*) and γ-amino butyric acid (GABA) (Schousboe *et al.*, 1977*b*) uptake, and glutamine synthetase activity (Hallermeyer *et al.*, 1981; Martinez-Hernandez *et al.*, 1977). The malignancy-associated properties investigated are general properties exhibited by many neoplastic cell types. Increased plasminogen activator levels associated with brain tumours have previously been reported (Tucker *et al.*, 1978; Hince & Roscoe, 1978) and the endothelial proliferation and neovascularisation associated with central nervous system tumours is well established (Feigin *et al.*, 1958; Gough, 1940; Krylova, 1973 and Pena & Feiter, 1973).

A number of phenotypic changes are known to accompany the transition from exponential growth to plateau phase in culture. In B16 melanoma cells, for example, tyrosinase activity reaches a maximum level soon after confluence has been reached (Wade & Burkart, 1978). Similarly, accumulation of S100 protein (Pfeiffer *et al.*, 1970), nerve growth factor (Schwartz *et al.*, 1977) and glycerol phosphate dehydrogenase (Bennett *et al.*, 1977) has been reported in $C_6$ cultures following confluence. These observations support the hypothesis that increased expression of differentiation occurs at high cell density. In this investigation the relationship between cell density and expression of GFAP and high affinity GABA uptake was examined. The effect of changing glioma cell density on plasminogen activator activity, a property which inversely correlated with biochemical differentiation in these cells, was also investigated.

Correspondence: R.I. Freshney.

*Present address: MRC Virology Unit, Inst. of Virology, Church St., Glasgow G11 5JR.

Received 20 October 1983; accepted 21 November 1983.

© The Macmillan Press Ltd., 1984

### Materials and methods

### *Tissue culture*

Cell cultures were derived from anaplastic astrocytomas (Grades III and IV Kernohan and Sayre histological grading), normal adult *post-mortem* brain and foetal brain, by fine dissection followed by dissociation in collagenase (CLS grade, Worthington 200 u ml$^{-1}$) for 24–48 h at 37°C. After removal of collagenase, tissue fragments were incubated in growth medium (Ham's F10 with 20 mM HEPES and 10% foetal calf serum, Flow Laboratories). Cultures were fed with fresh growth medium every few days and routinely subcultured with 0.25% trypsin (Gibco-Europe Ltd).

### *Glial fibrillary acidic protein (GFAP)*

Cells were washed with PBS (without $Ca^{2+}$ and $Mg^{2+}$) (PBSA) and fixed in cold acetone for 20 sec or methanol for 10 min at room temperature. The fixed cell preparations were stained for GFAP by the direct immunoperoxidase method using a 1:300 dilution of rabbit anti-GFAP (Palfreyman *et al.*, 1979).

### *Amino acid uptake*

The velocity of uptake of various concentrations of amino acids was determined by incubating for 20 or 40 min respectively at 37°C with a range of glutamic acid and GABA concentrations from 25 $\mu$M to 1 mM in Hank's balanced salts solution (HBSS) (Flow Laboratories) containing vitamins (Flow Laboratories) and 0.1% glucose. [$^3$H]-amino acids (L-[G-$^3$H] glutamic acid, 20–40 Ci mmol$^{-1}$ and 4-amino-n-[2,3-$^3$H] butyric acid, 50–70 Ci mmol$^{-1}$) (Radiochemical Centre, Amersham) were added at 5 $\mu$Ci ml$^{-1}$. Over the period of incubation for each amino acid the uptake was linear. Extracellular amino acids were removed by 3 washes with PBSA and the intracellular amino acids extracted in cold 10% trichloroacetic acid (TCA). The TCA extract was dissolved in Instagel scintillation fluid (Packard) and the [$^3$H] content determined by liquid scintillation counting. The velocity of uptake (Vi) was calculated and expressed as moles amino acids taken up per minute per million cells. Lineweaver-Burk, double reciprocal plots of Vi$^{-1}$ against [amino acid]$^{-1}$ were constructed for each cell line.

### *β-alanine sensitive GABA uptake*

The biphasic nature of double reciprocal GABA uptake by glial cells is indicative of the existence of a dual affinity mechanism of uptake. The higher affinity uptake process is completely inhibited by 2 mM β-alanine, shown in Figure 1 for the foetal brain-derived cell line NFF. Inhibition of 25 $\mu$M GABA uptake by 2 mM β-alanine has been used to quantitate high affinity GABA uptake.

**Figure 1** Lineweaver-Burk plots for the uptake of GABA into post-confluent NFF cells in the presence and absence of 2 mM β-alanine. Duplicate values are shown. Vi units = 10$^{-12}$ mol min$^{-1}$ 10$^6$ cells$^{-1}$. (GABA) units = mM (-----) with β-alanine; (——) without β-alanine.

### *Glutamine synthetase*

Cells grown to high density were harvested by trypsinisation, washed twice with PBSA and stored as a frozen pellet, Glutamine synthetase activities were determined by the procedure described by Reif-Lehrer (1971).

### *Plasminogen activator (PA)*

A modification of the chromogenic assay developed by Whur *et al.* (1980) was used to determine PA activities. Cells were washed 3 times with PBSA and incubated with a solution containing HBSS (without phenol red) (Flow Laboratories), glucose (0.1%) and vitamins, 1 mM chromogenic substrate S-2302 (Kabivitrum), 1 caseine unit ml$^{-1}$ plasminogen (Kabivitrum) and 0.15 mg ml$^{-1}$ poly-D-lysine (Sigma). The assay was a two step process using H-D-Propyl-L-phenyl- alanyl-L-arginine-p-nitroanilide (S-2302) as chromogenic substrate. The assay was terminated after 2 h with the addition of 5% acetic acid and the $OD_{405}$ corrected for cell number and endogenous plasmin in the plasminogen preparation. The proteolytic enzyme urokinase (Leo Laboratories) was used as a standard and PA activities were expressed as Plough units ml$^{-1}$ equivalents of urokinase 10$^6$ cells$^{-1}$.

*Angiogenesis*

*Extract preparation* Intracellular extracts of cell lines were prepared by repeated freezing and thawing of single cell suspensions followed by centrifugation at 48,000*g* for 30 min at 4°C. The supernatants were tested for angiogenic activity on the chick chorioallantoic membrane (CAM).

*CAM assay* The CAM was exposed through a small hole in the shell of a 9 day old chick embryo, and a small piece of Millipore filter (about 1 mm$^2$), soaked in protein extract, placed on the CAM. The hole was sealed with tape and the egg incubated at 37°C in a humidified incubator for 6 days. The CAM was dissected from the egg at day 15 and placed in formol saline. The extent of vasoproliferation was assessed using a dissection microscope.

### Results

The cell lines used in this investigation and their derivations are shown in Table I.

**Table I** Cell lines and their derivations

| *Cell line* | *Species* | *Tissue of origin* |
|---|---|---|
| NOR-F | Human adult | Brain (frontal lobe) |
| NOR-T | Human adult | Brain (temporal lobe) |
| GDU-T | Human adult | Brain (temporal lobe) |
| G-CCM | Human adult | Anaplastic astrocytoma |
| G-RAT | Human adult | Anaplastic astrocytoma |
| G-ATA | Human adult | Anaplastic astrocytoma |
| G-IJK | Human adult | Anaplastic astrocytoma |
| $C_6$ | Rat adult | Glioma |
| NFF | Human foetus | Brain |
| NFH | Human foetus | Brain |
| NFM | Human foetus | Brain |
| NFO | Human foetus | Brain |
| NFP | Human foetus | Brain |
| NFQ | Human foetus | Brain |

*Characterisation*

*Morphology* Cells cultured from normal brain had a flattened polygonal morphology forming continuous cell sheets at high density. The astrocytoma derived cell lines exhibited a range of morphological types consistent with the heterogeneous morphological phenotypes of a number of established glioma lines previously reported (Bigner *et al.*, 1981) and in general exhibited aneuploid karyotypes (Guner *et al.*, 1977). The foetal brain cultures were generally composed of two morphological cell types and were only successfully cultured for 8–12 generations.

*GFAP* GFAP was consistently visualised in only two of the glioma lines. G-CCM (Figure 2) was entirely GFAP positive while $C_6$ was 50–60% positive under standard culture conditions in subconfluent cultures. Of the remaining glioma lines G-IJK had only a minor component of GFAP positive cells and G-ATA and G-RAT lost any GFAP present in the primary cultures on subsequent subculturing. The foetal brain cultures were GFAP positive either partially, as in the case of NFM, NFH and NFP, or entirely as in the case of NFF and NFQ. The GFAP persisted in these cultures over the first few generations, during which time they were used experimentally. Cell lines derived from normal adult brain were entirely GFAP negative.

*High affinity amino acid uptake* The uptake kinetics of glutamic acid was investigated by Lineweaver-Burk analysis. Biphasic double reciprocal plots, as shown for the rat $C_6$ glioma (Figure 3a) are indicative of dual affinity mechanisms of glutamic acid uptake. The lower affinity mechanism has a Km of $4 \times 10^{-4}$ M and the higher afffinity of a Km of $3 \times 10^{-5}$ M. These values are comparable with those obtained by Logan & Snyder (1971) for low and high affinity glutamate uptake into homogenates and slices of rat cerebral cortex. The double reciprocal plot for GMS brain derived endothelial cells was monophasic, with only the low affinity (Km of $2.5 \times 10^{-4}$ M) mechanism of uptake evident (Figure 3b).

The specificity of high affinity glutamic acid uptake with respect to cell type is shown in Table II. Normal brain derived, glioma and melanoma cell lines were able to take up glutamic acid by

**Table II** Specificity of dual affinity glutamic acid uptake

| *Cell line* | *Type* | *Double reciprocal plot* | |
|---|---|---|---|
| | | *Monophasic* | *Biphasic* |
| NOR-F | Normal brain | | + |
| GDU-T | Normal brain | | + |
| G-CCM | Glioma | | + |
| G-IJK | Glioma | | + |
| G-ATA | Glioma | | + |
| G-RAT | Glioma | | + |
| $C_6$ | Glioma | | + |
| NFF | Foetal brain | | + |
| NFQ | Foetal brain | | + |
| GMS | Endothelial | + | |
| MRC-5 | Foetal fibroblasts | + | |
| 3T3 | Mouse fibroblasts | + | |
| FHI-4 | Foetal intestinal | + | |
| M-BRO | Melanoma | | + |
| M-AVO | Melanoma | | + |

**Figure 2** G-CCM glioma culture positively stained for GFAP by indirect immunoperoxidase. Scale bar 50 $\mu$M.

**Figure 3** Lineweaver-Burk plots for the uptake of glutamic acid into post-confluent cultures of (a) $C_6$ rat glioma cells and (b) GMS endothelial cells. Duplicate values are shown. Vi units = nmoles min$^{-1}$ $10^6$ cells$^{-1}$. [Glutamic acid] units = mM $Km_1$ = low affinity uptake; $Km_2$ = higher affinity uptake.

both low and high affinity processes, whereas endothelial cells, human and mouse fibroblasts and foetal human intestinal cells possessed only the low affinity mechanism of uptake.

The uptake of GABA was studied in a similar manner to that described for glutamic acid. The biphasic Lineweaver-Burk plot for GABA uptake by G-CCM, under standard culture conditions is shown in Figure 4. The Km value of $3.2 \times 10^{-5}$ M for high affinity uptake is similar to the range of Km $1.3$–$3.0 \times 10^{-5}$ M previously reported for various glial tumours maintained in tissue culture (Iversen & Kelly, 1975). Some cell lines did not express high affinity GABA uptake constitutively and required induction by a combination of steroid ($\beta$- or dexa-methasone) at 10 $\mu$g ml$^{-1}$ and dibutyryl cyclic AMP at 0.1 mM for several days. The

**Figure 4** Lineweaver-Burk plot for the uptake of GABA into post-confluent G-CCM cells. Duplicate values are shown. Vi units = $10^{-11}$ mol min$^{-1}$ $10^6$ cells$^{-1}$. [GABA] units = mM.

**Table III** Specificity of dual affinity GABA uptake

| *Cell line* | *Type* | *Inducer* | *Double reciprocal plot* | |
|---|---|---|---|---|
| | | | *Monophasic* | *Biphasic* |
| NOR-F | Normal brain | — | | + |
| NOR-T | Normal brain | — | + | |
| NOR-T | Normal brain | dbcAMP+DX | | + |
| GDU-T | Normal brain | — | + | |
| GDU-T | Normal brain | dbcAMP+DX | | + |
| G-CCM | Glioma | — | | + |
| $C_6$ | Rat glioma | — | | + |
| G-RAT | Glioma | — | + | |
| G-RAT | Glioma | dbcAMP+DX | | + |
| G-IJK | Glioma | — | + | |
| G-IJK | Glioma | dbcAMP+DX | | + |
| G-ATA | Glioma | — | + | |
| G-ATA | Glioma | dbcAMP+DX | + | |
| NFF | Foetal brain | — | | + |
| NFQ | Foetal brain | — | | + |
| M-ERS | Melanoma | — | + | |
| M-ERS | Melanoma | dbcAMP+DX | + | |
| MRC-5 | Fibroblasts | dbcAMP+DX | + | |
| GMS | Endothelia | dbcAMP+DX | + | |

specificity of high affinity GABA uptake and the induction requirements are shown in Table III.

*Glutamine synthetase* The GS activities of extracts from cells grown under standard culture conditions are shown in Table IV. The normal adult cells had relatively low levels of enzyme, foetal lines 2–3 fold greater activity than the normal adult lines and the gliomas had variable GS activities with G-CCM having the highest level detected.

**Table IV** Glutamine synthetase specific activities

| *Cell line* | *GS specific activity n moles product formed* $min^{-1}$ *mg protein*$^{-1}$ |
|---|---|
| GDU-T | 11.2 |
| NOR-T | 10.0 ± 5.0 |
| G-CCM | 45.0 ± 15.0 |
| G-IJK | 24.0 ± 10.6 |
| G-ATA | 10.0 ± 4.7 |
| G-RAT | 22.0 ± 12.0 |
| $C_6$ | 4.0 ± 0.5 |
| NFH | 36.0 ± 27.0 |
| NFO | 32.5 ± 8.0 |
| NFP | 22.8 ± 8.3 |

*Plasminogen activator* (*PA*) The production of plasminogen activator by cells was determined over a period of 2 h (Figure 5). PA production by two astrocytoma cell lines not included in the general study, G-JPT and G-VAG are also shown. In general the normal adult cell lines had lower levels of activity than the GFAP negative gliomas. The GFAP positive glioma lines G-CCM and $C_6$ had very low levels of plasminogen activator.

*Angiogenesis* Intracellular extracts were prepared as described in **Materials and methods** and tested for angiogenic activity on the chick CAM. An attempt was made to grade the responses. The negative response of bovine serum albumin was designated as 0, the almost complete surrounding of WRC-256 extract soaked filters by radial blood vessels was designated as 4 and intermediate responses were designated as 1, 2 or 3. Figure 6 shows CAMs on which small pieces of Millipore filters soaked in 2 mg $ml^{-1}$ protein extracts of NOR-F, $C_6$ and G-RAT cells respectively have been implanted for 6 days. These samples showed a gradation in response from no obvious vasoproliferation for NOR-F (Figure 6a) to considerable activity for G-RAT (Figure 6c) and were designated as 0, 2 and 4 respectively. The means and standard deviations of angiogenic responses are shown in Figure 7. Extracts of the glioma lines clearly induced angiogenesis while normal adult and foetal lines showed little activity. The large standard deviations evident in Figure 7 were, in part, the result of unavoidable variation in the site of implantation of the extract-soaked filter on the CAM. In particular, the proximity of the filter to

**Figure 5** Plasminogen activator (PA) activities of a variety of GFAP negative astrocytomas, GFAP positive gliomas and cell lines derived from normal adult and foetal brain. PA assays were carried out by the modified procedure of Wuhr *et al.* (1980) as described in **Materials and methods**. Duplicate values are shown. Units of PA activity = Plough units $10^6$ cells$^{-1}$.

major blood vessels affected the magnitude of the angiogenic response. The subjective nature of the gradation introduced further variation to the assay.

A composite table showing the differentiated and malignancy-associated properties attributable to the normal adult, foetal and malignant brain derived cell lines is presented in Table V.

*Density dependent control of phenotypic expression* The effect of increasing monolayer cell density on GFAP in cultures of rat $C_6$ glioma is shown in Figure 8. As $C_6$ approached confluence, at around $10^5$ cells cm$^{-2}$, there was a dramatic increase in the proportion of cells expressing GFAP from <50% to ~75%. The rat $C_6$ was the only cell line used in this investigation which exhibited density dependent induction of GFAP. The human glioma and normal adult brain lines were either 100% GFAP positive regardless of cell density or were predominantly or entirely GFAP negative. There was no visually detected increase in the number of GFAP positive cells with increasing cell density in any of these cell lines.

A similar effect to that observed for GFAP in $C_6$ cultures was demonstrated for high affinity GABA uptake by a number of cell lines. β-Alanine sensitive GABA uptake by NOR-F, the normal human adult brain derived cell line, and $C_6$ at various cell densities, is presented in Figures 9a and b respectively. This increase in β-alanine sensitive GABA uptake at high density was observed for all the normal adult brain and astrocytoma cultures investigated. The high density phase of culture growth is characterised by reduced proliferation and increased formation of cell–cell contacts. In order to distinguish between these growth was inhibited by withdrawing serum from exponentially growing cultures of NOR-F cells, thus inducing cytostasis without increased intercellular contact formation. The β-alanine sensitive GABA uptake

**Figure 6** Angiogenic responses on the chick CAM of intracellular extracts of (a) NOR-F (normal brain derived), (b) $C_6$ (rat glioma) and (c) G-RAT (anaplastic astrocytoma) cell lines. The variation in the extent of induced vasoproliferation induced by different cell lines is evident.

**Figure 7** Semi-quantitative gradation of the angiogenic responses of extracts prepared from various cell lines as measured by vasoproliferation on the CAM. The mean scores and standard deviations of 7–10 replicates are shown.

**Table V** Composite table

| | Cell line | GFAP | High affinity glut. uptake | High affinity GABA uptake | GS activity | PA | Angiogenic response |
|---|---|---|---|---|---|---|---|
| Normal brain | NOR-F | – | + | c+ | | | |
| | NOR-T | – | | i+ | l | l | – |
| | GDU-T | – | + | i+ | l | l | – |
| Glioma | $C_6$ | + | + | c+ | l | l | + |
| | G-CCM | + | + | c+ | h | l | + |
| | G-RAT | – | + | i+ | h | int | + |
| | G-ATA | – | + | – | l | h | + |
| | G-IJK | +/– | + | i+ | h | int | + |
| Foetal brain | NFF | + | + | c+ | | | |
| | NFH | + | | | h | int | |
| | NFM | + | | | | | |
| | NFO | + | | | | | |
| | NFP | + | | | h | | |
| | NFQ | + | + | c+ | | | |

– negative; + positive; +/– a few positive cells; c+ constitutive; i+ inducible.
GS activity: h high > 20 enzyme units.
l low < 12 enzyme units.
PA activity: l low.
int intermediate.
h high.

**Figure 8** $C_6$ rat glioma cultures were grown in 75 $cm^2$ flasks and stained for GFAP by immunoperoxidase at the densities indicated. Between 200–500 cells were scored and the % GFAP positive cells determined. Each point is the mean and standard deviation of 10–20 replicate fields from duplicate samples.

**Figure 9** Semi-logarithmic plots of (a) NOR-F (normal adult brain) and (b) $C_6$ rat glioma mean cell densities against day of growth in 24-well plates. $\beta$-Alanine sensitive GABA (BASG) uptake measurements were made at the time points indicated as described in **Materials and methods**. BASG points are the means and standard deviations of 4 replicate measurements. Units of BASG uptake $= 10^{-12}$ mol min$^{-1}$ $10^6$ cells$^{-1}$.

under these conditions, at a density of $5.2 \times 10^4$ cells cm$^{-2}$ was $1.30 \times 10^{-12}$ mol min$^{-1}$ $10^6$ cells$^{-1}$. This compares with a value of $1.33 \times 10^{-12}$ mol min$^{-1}$ $10^6$ cells$^{-1}$ for cells at this density under standard culture conditions, thus showing that cytostasis alone was not sufficient to induce the increase in $\beta$-alanine sensitive GABA uptake observed at high density. Cell viability, as determined by trypan blue dye exclusion, was unaffected by serum withdrawal for several days.

Plasminogen activator activity in gliomas decreased with increasing cell density. This applied to all the cell lines investigated although the effect was most obvious in cell lines exhibiting high plasminogen activator activities (Figures 10a and b). After confluence had been reached very little further decrease in plasminogen activator activity was observed.

**Figure 10** Semi-logarithmic plots of (a) G-ATA anaplastic astrocytoma and (b) $C_6$ rat glioma mean cell densities against day of growth in 24-well plates The cells were assayed for plasminogen activator (PA) activity at the times indicated. PA activities are the means and standard deviations of 4 replicate measurements. Units of PA activity = Plough units $10^6$ cells$^{-1}$.

### Discussion

Cell lines derived from histologically similar anaplastic astrocytomas showed considerable heterogeneity of phenotypic expression. This reflects the heterogeneous nature of cells in malignant astrocytomas and the inevitable selection of cells most suited to growth in culture. The cell lines used in this study exhibited a range of morphologies and states of biochemical differentiation from highly differentiated, in the case of G-CCM to undifferentiated, in the case of G-ATA. There was no obvious correlation between cell morphology and differentiation.

The astrocytic marker protein, GFAP, was present in cultures derived from 12–16 weeks post-conception foetal brain and in the gliomas G-CCM and $C_6$, demonstrating that highly differentiated astroglial cells are able to grow and divide in culture. In the foetal and tumour situations, it appears that the normal relationship between terminal differentiation and proliferation may be disturbed. Although GFAP can be detected in cells in most astrocytic gliomas, only the most morphologically differentiated cells express it, while the more primitive and anaplastic cells do not (Velasco *et al.*, 1980; Eng & Rubinstein, 1978). Bigner *et al.* (1981) reported that only 2/15 established glioma cell lines had readily detectable levels of GFAP, and it has also been previously shown that many astrocytoma cell lines are entirely GFAP negative after repeated subculturing (Vivard *et al.*, 1978).

High affinity glutamic acid uptake was expressed by all the brain-derived cell lines tested as well as by melanoma cell lines, which, like glial cells, are derived from the neuroectoderm. This implied that high affinity glutamic acid uptake was perhaps specific to cells of neuroectodermal origin, rather than glial cells. The non-neuroectodermally derived control cell lines, did not express this property. High affinity GABA uptake appears to be a more specific marker for differentiation in glial cells. Melanoma cells and the non-neuroectodermal control cells did not express high affinity GABA uptake, even after treatment with the inducers dibutyryl cyclic AMP and dexamethasone.

The GFAP-positive foetal and glioma cultures expressed high affinity GABA uptake constitutively, confirming their differentiated phenotype. GABA uptake was induced in two of the predominantly non-GFAP expressing glioma lines, G-RAT and G-IJK, by a combination of dibutyryl cyclic AMP and dexamethasone. G-ATA was not inducible. Of the normal adult cell lines, one was constitutive for high affinity GABA uptake and two were inducible by the above combination. The last observation together with the absence of GFAP from these cell lines implied an undifferentiated status for the normal adult cells. One possibility is that these are precursor glial cells, selected from a precursor population in the brain during the initial culturing procedure.

The highest levels of glutamine synthetase (GS) activity were attributed to the foetal cultures and the GFAP positive human glioma G-CCM. The rat $C_6$ glioma had a low GS specific activity, perhaps a consequence of the stem cell nature of the $C_6$ tumour and its consequent ability to express astrologlial and oligodendroglial properties simultaneously (McCormick & Wallace, 1982). With the exception of $C_6$ the lowest GS activity was attributed to the undifferentiated G-ATA.

Increased plasminogen activator has been correlated with expression of the malignant phenotype in a number of experimental systems (Mak *et al.*, 1976; Pollack *et al.*, 1974; Rifkin *et al.*, 1974) and is widely believed to have a role in the growth and spread of tumours. Pearlstein *et al.* (1976), however, reported that high levels of fibrinolytic activity can be demonstrated in some tumour cells, but not in all. In this series of experiments, the less well differentiated astrocytoma cell lines, in particular G-ATA, exhibited high levels of PA. The GFAP positive gliomas G-CCM and $C_6$ had very low levels of activity and the cell lines of intermediate differentiation status, G-RAT and G-IJK had intermediate PA activities. It thus appeared that an inverse relationship existed between PA and the biochemical differentiation status of the glioma lines tested.

There was no apparent relationship between differentiation and angiogenic activity as determined by the ability of intracellular extracts to stimulate vasoproliferation on the chick CAM. Glioma cell lines exhibited an angiogenic response regardless of their expression of differentiated properties, perhaps reflecting the absolute requirement for the cells of solid tumours to be able to induce proliferation of the host vasculature. Extracts of the normal brain-derived cultures did not elicit a significant angiogenic response.

A number of alterations in cell behaviour accompany the transition from subconfluent to confluent cell densities, the most striking of which is a reduction in the growth fraction (Westermark, 1973). In general transformed cells are less sensitive to restriction of growth at high density, e.g. glioma cells have a higher growth fraction in dense culture and reach a higher saturation density than normal glia (Westermark, 1973). In particular there is very little reduction in the proliferative capacity of $C_6$ cells at confluence, when differentiation is markedly increased. This implies that cytostasis is not the primary event triggering the increased production of differentiation markers. As cells approach confluence the amount of membrane contacts between cells also increases. In the rat $C_6$ glioma at confluence the observed increase in GFAP is consistent with the accumulation of other differentiated products, such as S-100 protein, at this point in culture growth. Pfeiffer *et al.* (1970) demonstrated that $C_6$ cells isolated from one another in suspension did not accumulate S100 protein when proliferation was stopped with metabolic inhibitors. Hence an important role of cell–cell contacts was thus implied in the expression of a differentiated product.

The exact nature of the membrane contacts involved in the control of phenotypic expression is unknown although $C_6$ cells are known to form gap functions in culture. It might be the increase in the number of communication channels between cells at confluence, allowing metabolic sharing, that is responsible for the increase in differentiated properties. Alternatively the effect might be the result of cell interactions at specific membrane recognition sites. With the exception of one human glioma line, the normal adult brain and astrocytoma derived cell lines entirely or predominantly lacked GFAP. Increasing cell density did not induce expression of the astrocytic marker in these lines.

A role for increased membrane contacts is also implied for the observed increase in high affinity GABA uptake by normal adult brain and glioma cells. In this case cytostasis, induced by serum withdrawal prior to confluence, may have been necessary but was not sufficient for the induction of differentiation.

The cellular activity of plasminogen activator, a property associated with malignant brain tissue, decreased with increasing cell density. A steady-state level of activity was reached as cells approached confluence.

In conclusion, the results of this investigation imply that in gliomas the expression of differentiated properties and expression of plasminogen activator may be inversely related. The ability to induce neovascularisation is unrelated to differentiation. The expression of differentiated properties and expression of plasminogen activator appear to respond in an opposite manner to changes in monolayer cell density.

The authors are grateful to SERC (Case studentship to MCF), Beechams Pharmaceuticals, Elspeth McKenzie Memorial Trust and the Scottish Home and Health Department (PFTV) for support. The skilled technical assistance of Mr A. Wilson was greatly appreciated.

**References**

BENNETT, K., McGINNIS, J.F. & DEVELLIS, J. (1977). Reversible inhibition of the hydrocortisone induction of glycerol phosphate dehydrogenase by cytochalasin B in C6 cells. *J. Cell Physiol.*, **93**, 247.

BIGNER, D.D., BIGNER, S.H., PONTEN, J. & 6 others. (1981). Heterogeneity of genotypic and phenotypic characteristics of fifteen permanent cell lines derived from human gliomas. *J. Neuropathol. Exp. Neurol.*, **40**, 201.

ENG, L.F. & RUBINSTEIN, L.J. (1978). Contribution of immunohistochemistry to diagnostic problems of human cerebral tumours. *J. Histochem. Cytochem.*, **26**, 513.

ENG, L.F., VANDERHAEGHEN, J.J., BIGNAMI, A. & GARSTLE, B. (1971). An acidic protein isolated from fibrous astrocytes. *Brain Res.*, **28**, 351.

FEIGIN, I., ALLEN, L.B., LIPKIN, L. & GROSS, S.W. (1958). The endothelial hyperplasia of the cerebral blood vessels with brain tumours, and its sarcomatous transformation. *Cancer*, **11**, 264.

GOUGH, J. (1940). The structure of the blood vessels in cerebral tumours. *J. Pathol. Bacteriol.*, **51**, 23.

GUNER, M., FRESHNEY, R.I., MORGAN, D., FRESHNEY, M.G., THOMAS, D.G.T. & GRAHAM, D.I. (1977). Effects of dexamethasone and betamethasone on *in vitro* cultures from human astrocytoma. *Br. J. Cancer*, **35**, 439.

HALLERMEYER, K., HARMENING, C. & HAMPRECHT, B. (1981). Cellular localisation and regulation of glutamine synthetase in primary cultures of brain cells from newborn mice. *J. Neurochem.*, **37**, 43.

HINCE, T.A. & ROSCOE, J.P. (1978). Fibrinolytic activity of cultured cells derived during ethylnitrosourea-induced carcinogenesis of rat brain. *Br. J. Cancer*, **37**, 424.

IVERSEN, L.L. & KELLY, J.S. (1975). Uptake and metabolism of γ-amino butyric acid by neurones and glial cells. *Biochemical Pharmacol.*, **24**, 933.

KRYLOVA, N.V. (1973). Endothelium characteristics of blood vessels in neoplasms. *Bibl. Anat.*, **12**, 497.

LOGAN, W.J. & SNYDER, S.H. (1971). Unique high affinity uptake systems for glycine, glutamic and aspartic acids in central nervous tissue of the rat. *Nature*, **234**, 297.

MAK, T.W., RUTLEDGE, G. & SUTHERLAND, D.J. (1976). Androgen-dependent fibrinolytic activity in a murine mammary carcinoma (Shionogi SC:115 cells) *in vitro*. *Cell*, **7**, 223.

MARTINEZ-HERNANDEZ, A., BELL, K.P. & NORENBERG, M.D. (1977). Glutamine synthetase: Glial localisation in brain. *Science*, **195**, 1356.

McCORMICK, D. & WALLACE, I. (1983). Simultaneous expression of astrocytic and oligodendrocytic features in recloned cell lines from the rat C6 glioma. *Abstract, British Glioma Group 3rd Annual Meeting, Bristol*, 1983.

PALFREYMAN, J.W., THOMAS, D.G.T., RATCLIFFE, J.G. & GRAHAM, D.I. (1979). GFAP: Purification from a human fibrillary astrocytoma, development and validation of a radioimmunoassay for GFAP-like immunoactivity. *J. Neurol. Sci.*, **41**, 101.

PEARLSTEIN, E., HYNES, R.O., FRANKS, L.M. & HEMMINGS, V.J. (1976). Surface proteins and fibrinolytic activity of cultured mammalian cells. *Cancer Res.*, **36**, 1475.

PENA, C.E. & FEITER, R. (1973). Ultrastructure of a composite glioma-sarcoma of the brain. *Acta. Neuropathol.*, **23**, 90.

PFEIFFER, S.E., HERSCHMAN, M.R., LIGHTBODY, J. & SATO, G. (1970). Synthesis by a clonal line of rat glial cells of a protein unique to the nervous system. *J. Cell. Physiol.*, **75**, 329.

POLLACK, R., RISSER, R., CONLON, S. & RIFKIN, D. (1974). Plasminogen activator production accompanies loss of anchorage regulation in transformation of primary rat embryo cells by simian virus 40. *Proc. Natl Acad. Sci.*, **71**, 4792.

REIF-LEHRER, L. (1971). Actinomycin-D enhancement of glutamine synthetase activity in chick embryo retinas cultured in the presence of cortisol. *J. Cell Biol.*, **51**, 303.

RIFKIN, D.B., LOEB, J.N., MOORE, G. & REICH, E. (1974). Properties of plasminogen activators formed by neoplastic human cell cultures. *J. Exp. Med.*, **139**, 1317.

SCHOUSBOE, A., SVENNEBY, G. & HERTZ, L. (1977*a*). Uptake and metabolism of glutamate in astrocytes cultured from dissociated mouse brain hemispheres. *J. Neurochem.*, **29**, 999.

SCHOUSBOE, A., WU, P.H., HERTZ, L. & FEDEROFF, S. (1977*b*). GABA formation, uptake. release and metabolism in cultured normal astrocytes. *Trans. Am. Soc. Neurochem.*, **8**, 248.

SCHWARTZ, J.P., CHUANG, D.M. & COSTA, E. (1977). Regulation by isoproterenol of nerve growth factor levels in C6 glioma. *Trans. Am. Soc. Neurochem.*, **8**, 141.

TUCKER, W.S., KURSCH, W.M., MARTINEZ-HERNANDEZ, A. & FINK, L.M. (1978). *In vitro* plasminogen activator activity in human brain tumours. *Cancer Res.*, **38**, 297.

VELASCO, M.E., DAHL, D., ROESSMANN, U. & GAMBETTI, P. (1980). Immunohistochemical localization of glial fibrillary acidic protein in human glial neoplasms. *Cancer*, **45**, 484.

VIVARD, M.N., GIRARD, N., CHAUZY, C. & 4 others. (1978). Disparition de la proteine gliofibrillaire (GFA) aucours de la culture de cellules de glioblastomes. *Compte Rendue Acad. Sci.*, **286**, 1837.

WADE, D.R. & BURKART, M.E. (1978). The role of adenosine 3′,5′-cyclic monophosphate in the density dependent regulation of growth and tyrosinase activity of B-16 melanoma cells. *J. Cell Physiol.*, **94**, 265.

WESTERMARK, B. (1973). The deficient density-dependent growth control of human malignant glioma cells and virus-transformed glia-like cells in culture. *Int. J. Cancer*, **12**, 438.

WHUR, P., MAGUIDA, M., BOSTON, J., LOCKWOOD, J. & WILLIAMS, D.C. (1980). Plasminogen activator in cultured Lewis Lung Carcinoma cells measured by chromogenic substrate assay.*Br. J. Cancer*, **42**, 305.